# Are there conserved biosynthetic genes in lichens? Genome-wide assessment of terpene biosynthetic genes suggests ubiquitous distribution of the squalene synthase cluster

**DOI:** 10.1186/s12864-024-10806-0

**Published:** 2024-10-07

**Authors:** Garima Singh, Anna Pasinato, Alejandra López-Chicheri Yriarte, David Pizarro, Pradeep K. Divakar, Imke Schmitt, Francesco Dal Grande

**Affiliations:** 1 Department of Biology, University of Padova, Via U. Bassi, 58/B, 35121 Padua, Italy; 2 Botanical Garden of Padova, University of Padova, Padua, Italy; 3 Department of Pharmacology, Pharmacognosy and Botany, Faculty of Pharmacy, Complutense University of Madrid (UCM), Madrid, 28040 Spain; 4 Senckenberg Biodiversity and Climate Research Centre (SBiK-F), Frankfurt Am Main, 60325 Germany; 5 Department of Biosciences, Institute of Ecology Evolution and Diversity, Goethe UniversityFrankfurt,, Max-Von-Laue-Str. 13, Frankfurt am Main, 60438 Germany; 6 LOEWE Center for Translational Biodiversity Genomics (TBG), Frankfurt Am Main, 60325 Germany

**Keywords:** BiG-SCAPE, Secondary metabolites, Genome mining, Ergosterol, AntiSMASH, Lecanoromycetes

## Abstract

Lichen-forming fungi (LFF) are prolific producers of functionally and structurally diverse secondary metabolites, most of which are taxonomically exclusive and play lineage-specific roles. To date, widely distributed, evolutionarily conserved biosynthetic pathways in LFF are not known. However, this idea stems from polyketide derivatives, since most biochemical research on lichens has concentrated on polyketide synthases (PKSs). Here, we present the first systematic identification and comparison of terpene biosynthetic genes of LFF using all the available Lecanoromycete reference genomes and 22 de novo sequenced ones (111 in total, representing 60 genera and 23 families). We implemented genome mining and gene networking approaches to identify and group the biosynthetic gene clusters (BGCs) into networks of similar BGCs. Our large-scale analysis led to the identification of 724 terpene BGCs with varying degrees of pairwise similarity. Most BGCs in the dataset were unique with no similarity to a previously known fungal or bacterial BGC or among each other. Remarkably, we found two BGCs that were widely distributed in LFF. Interestingly, both conserved BGCs contain the same core gene, i.e., putatively a squalene/phytoene synthase (SQS), involved in sterol biosynthesis. This indicates that early gene duplications, followed by gene losses/gains and gene rearrangement are the major evolutionary factors shaping the composition of these widely distributed SQS BGCs across LFF. We provide an in-depth overview of these BGCs, including the transmembrane, conserved, variable and LFF-specific regions. Our study revealed that lichenized fungi do have a highly conserved BGC, providing the first evidence that a biosynthetic gene may constitute essential genes in lichens.

**Supplementary Information:**

The online version contains supplementary material available at 10.1186/s12864-024-10806-0.

## Background

Lichens, symbiotic association between a fungus and one/more photosynthetic partners (algae/cyanobacteria), are prolific producers of structurally diverse secondary metabolites that play essential roles in organism survival, defense and ecological interactions [1–3]. Among the approximately 1000 lichen metabolites reported from LFF, to our knowledge, none has been shown to be ubiquitously distributed [4–7]. Consequently, no secondary metabolite pathway has been shown to be evolutionarily conserved in fungi. This notion, however, is mostly based on the studies on polyketide derivatives—the most well-studied lichen compounds in terms of bioactivity, chemistry and pathways (e.g., olivetoric acid, [8], e.g., grayanic acid, [9], usnic acid [10–12], and gyrophoric acid [13]). Some PKS derivatives are reported to be secreted by several LFF and the concerned biosynthetic gene clusters (BGCs) have a relatively wider taxonomic distribution (e.g., atranorin [14] and anthraquinones in Teloschistales [15]). Nonetheless, the presence of ubiquitously present BGCs in lichenized fungi has not been reported. The evolutionarily conserved presence of a BGC would suggest an important functional role, shedding light on the significance of secondary metabolites for lichens.

Terpenes are an interesting class of biosynthetic compounds owing to their ecological and physiological importance in all domains of life, including bacteria [16–18], non-lichenized model fungi, and plants [19]. Terpene synthases play pivotal roles in the basic biological functions such as in metabolism, cell wall and membrane formation and in establishing symbiotic relationships [16, 18, 20]. In fact, certain terpene BGCs have been shown to be widely distributed across plants and animals [18, 21]. For this reason, they constitute an ideal candidate for being evolutionarily conserved.

Terpene biosynthetic gene clusters are the third largest class of BGCs in LFF after PKSs and NRPSs [11, 13, 15]. As typical of BGCs, they also exhibit collinear and proximate arrangements of genes involved in metabolite synthesis [22–25]. A terpene BGC typically consists of a terpene cyclase (TPC) as the core enzyme that forms the hydrocarbon backbones of terpenoids and a few accessory enzymes that modify the backbone terpene or are involved in the regulation of terpene synthesis or the transportation of the final product [23, 24, 26]. The most common tailoring enzymes associated with terpene BGCs are cytochrome P450 mono-oxygenases (CYP450), NAD(P) + , and flavin-dependent oxidoreductases. Although our understanding of the diversity and evolution of terpene BGCs in non-lichenized fungi has significantly advanced, exploration of the terpene biosynthetic genes in LFF remains uncharted. To our knowledge, all studies thus far have focused on terpene detection and its taxonomic significance for species identification [21, 27, 28], whereas large-scale metagenomic investigations of terpene biosynthetic gene clusters (BGCs) have never been carried out. This research gap could be predominantly attributed to the scarcity of publicly available genomic resources required for such investigations. The recent increase in the taxonomic coverage of LFF in genomic databases provides a premise for exploring the possibility of exploring the diversity and evolution of terpene BGCs in LFF [29, 30].

In this study, we aimed to answer the following questions: 1) What is the diversity of BGCs linked to terpene biosynthesis in LFF? 2) Are there any evolutionarily conserved BGCs related to terpene biosynthesis? 3) What are the evolutionary forces determining the organization of terpene BGCs in LFF?

## Methods

### Dataset, genome assembly and annotation

A total of 111 LFF were included in the study, comprising 102 Lecanoromycetes, four Eurotiomycetes and five Dothideomycetes fungi (Supplementary Material 1). The dataset includes all the Lecanoromycete reference genomes, one per species, available in NCBI until February 2023.

Twenty-seven genomes were de novo sequenced for this study using Illumina sequencing technology. To obtain the genome assemblies, the trimmed paired-end reads were assembled using MetaSPAdes [31] with default parameters, ensuring the suitability of k-mers (K21, K33, K55, and K77). To isolate the contigs of fungal-origin, the preliminary assemblies were subjected to BLASTX searches using DIAMOND [32] against a custom database. This database included protein sets from Archaea, Bacteria, Eukaryota, and Viruses from the NCBI non-redundant database (downloaded in August 2022), along with 150 complete fungal genomes and 20 algal genomes from JGI. Additionally, following four LFF genomes were used as reference for the taxonomic assignment; *Evernia prunastri* and *Pseudevernia furfuracea* genomes from Meiser et al. [33], and two de novo sequenced genomes from the axenic cultures of *Cetraria islandica* and *Parmelina carporrhizans*. The DIAMOND search results were then processed using MEGAN6 [34] for taxonomic binning (parameters: min-support = 1, min-score = 50, top-hit = 10%, no low complexity filtering). Contigs identified as Parmeliaceae were extracted, and genome statistics were generated using QUAST v. 4.3 [35]. Gene prediction and functional annotation on resulting genome assemblies were performed with funannotate [36]. Briefly, first the repetitive elements were masked in the assembled genomes. Gene prediction was then performed on the masked genomes using the gene predictor Augustus trained with BUSCO [37] and self-training gene predictor GeneMark-ES [38]. Functional annotation was carried out with InterProScan [39, 40], egg-NOG-mapper [41]and BUSCO [42, 43] ascomycota_odb10 models. Secreted proteins were predicted using SignalP [44] as implemented in the command funannotate ‘annotate’. Genome completeness was estimated using BUSCO (Benchmarking Universal Single-Copy Orthologs [42]) and the Ascomycota database.

### Species tree reconstruction

**Fig. 1:**
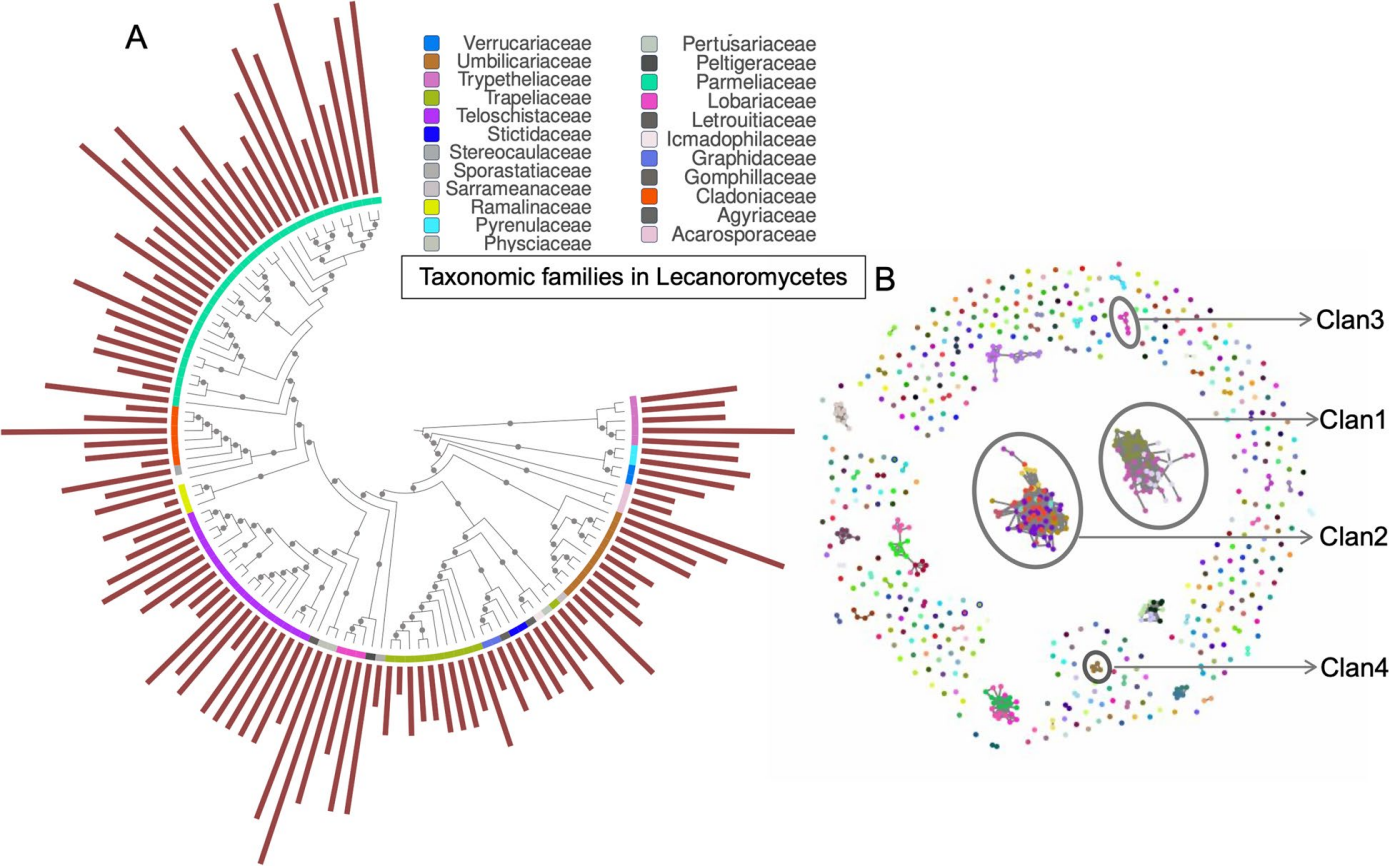
Frequency and diversity of terpene biosynthetic genes in lichens belonging to Lecanoromycetes and nine others belonging to Eurotiomycetes and Dothideomycetes. **A** Cladogram derived from the ML phylogenomic tree based on single-copy BUSCOs of 111 species, with grey circles on the branches representing >70% bootstrap support. The bars depict the number of terpene BGCs present in each taxon. Although the number of terpene BGCs varies across species, all lichens have at least one terpene-related BGC. **B** Terpene BGC network generated by BiG-SCAPE (raw-distance cutoff 0.6) showing the grouping of BGCs into gene cluster families and clans based on gene homology and BGC synteny. Each dot in the network represents a terpene BGC and lines connect similar BGC. Each BGC in the network is found in a different species, with no two BGCs in the same clan found in the same genome. Different colors refer to different gene cluster families (according to BiG-SCAPE). Most terpene BGCs are singletons with no connections, indicating their uniqueness and restricted taxonomic distribution. Two large terpene clans were detected (Clan1 and Clan2), represented by the crowded dots connected by lines, suggesting their widespread presence in LFF. In addition, two other clans were detected that had the same core gene as Clan1 and Clan2, a squalene synthase - Clan3 and Clan4

Species tree reconstruction was done by implementing the phylogenomic pipeline mentioned at https://github.com/mcmurtrs/Making-a-Phylogenetic-Tree-with-BUSCO-Genes. Briefly, single-copy BUSCOs were quality filtered and compared among species to extract those present in most species (a maximum of one sample missing). The resulting BUSCOs were then concatenated, and the concatenated sequences from all the species were aligned using MAFFT L-INS-I [45, 46] (multiple alignment using fast Fourier transform). Phylogenetic relationships were inferred from the alignment using maximum likelihood (ML) analysis as implemented in IQTree v1.5.5 [47] using auto substitution model selection and 1,000 bootstrap replicates. The model chosen was LG + G4m. The resulting tree was visualized using FigTree 1.3.1 [48] and annotated in iTOL (integrated tree of life, [49]) (Fig. 1A).

### BGC identification and clustering using automated genome mining software

Biosynthetic genes were predicted and annotated in all the genomes using the automated genome mining pipeline implemented in AntiSMASH (antibiotics & SM Analysis Shell, v7.0 [50]), which identifies BGCs based on probabilistic models (HMMs). The predominant class of BGCs identified by AntiSMASH includes those containing the following core genes: polyketide synthases (PKSs), non-ribosomal peptide synthases (NRPSs), terpenes, ribosomally-synthesized and post-translationally modified peptides (RiPPs), and hybrid BGCs. BGCs identified via AntiSMASH exhibit varying degrees of similarity to a characterized BGC present in the MIBiG repository (Minimum Information about a Biosynthetic Gene cluster) and to each other. MIBiG is a repository comprising standardized entries for experimentally validated BGCs of known function from different domains of life, e.g., bacteria, fungi and plants [51].

To compare gene sequences and BGCs identified via AntiSMASH and identify homologous and widely distributed BGCs, we used the biosynthetic gene similarity clustering and prospecting engine or the BiG-SCAPE program ([52], https://git.wageningenur.nl/medema-group/BiG-SCAPE). BiG-SCAPE builds sequence similarity networks for each BGC class. Within each network, similar BGCs are grouped into gene cluster families (GCFs) and two or more GCFs potentially encoding structurally similar compounds are grouped into clans. Each network (terpene, PKS or NRPS) therefore contained several GCFs and clans (Fig. 1B). The number of clans detected for a network also depends on the clustering threshold employed, with lower cut-offs implying a stricter clustering threshold, leading to fewer connections and vice versa. We generated the BGC network by applying raw distance cutoffs of 0.20, 0.4, 0.6, and 0.80. To prevent overestimation of potentially novel BGCs, we chose the network with a cutoff of 0.6. The analysis was performed by retaining singletons and using the PFAM (protein families, v37.0 (21,979 entries, 709 clans)) database [53].

The gene network of each biosynthetic class was inspected for the presence of widely distributed BGCs. Among the clans obtained for each network, the largest GCFs/clans were obtained for terpenes (represented by Clan1 and Clan2 in Fig. 1B). The core genes of the two clans were identified based on similarity to a characterized biosynthetic gene in the MIBiG repository. This analysis suggested that the core genes of both widely distributed BGCs are SQSs, which are involved in the synthesis of cholesterol/ergosterol. We then continued with in-depth analyses of the two terpene clans to identify them in silico and explore their diversity, homology and synteny across LFF.

The widely distributed terpene BGCs were not detected by BiG-SCAPE for some species. We first verified if this observation could simply be an artefact of sequencing technology or BGC prediction and clustering algorithms as the absence of otherwise conserved genes in a taxon indicates major evolutionary impacts. To fish out the other SQS BGCs in our dataset that did not group within Clan1 and Clan2, we implemented a twofold approach. First, we investigated whether there were other clans in the terpene network that had an SQS as the core gene. Second, we performed local BLAST using the SQSs of Clan1 and Clan2 as queries and searched them in a database composed of all the terpene synthases of the species in which no SQS was detected by BiG-SCAPE (using a 30% sequence similarity threshold). When no SQS was detected for a taxon in the database, we further validated the absence of the SQS in that taxon by aligning the raw sequencing reads with the Clan1 and Clan2 SQSs. If no reads aligned to the SQS of Clan1 or Clan2, this was considered evidence for the absence of this BGC in that taxon.

**Fig. 2:**
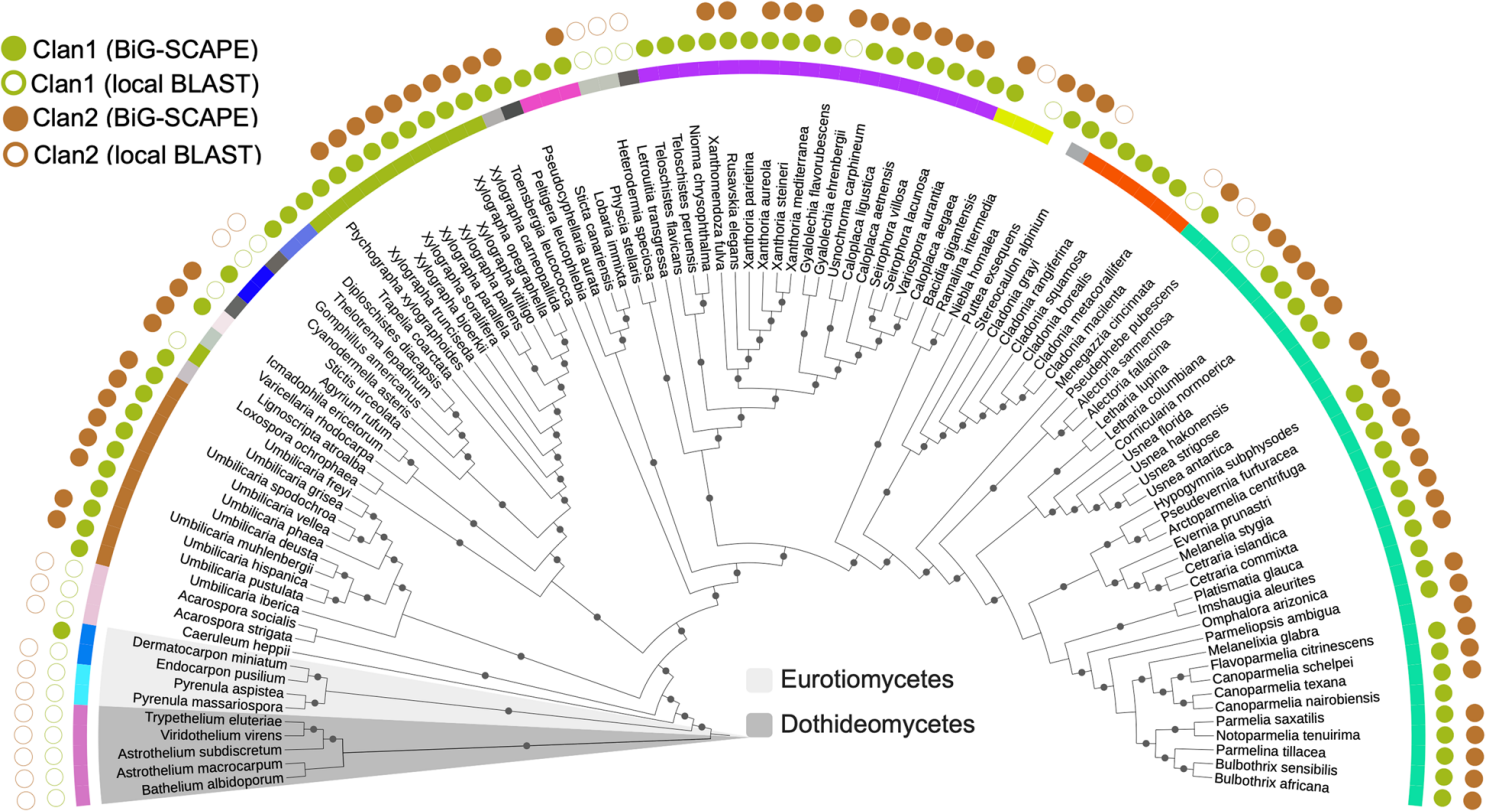
Phylogenetic distribution of the two conserved SQS BGC clans. A cladogram depicting the distribution of Clan1 and Clan2 in LFF, as shown in Fig. 1B,  and in the outgroup taxa. Dots on the branches indicate bootstrap support > 70. The empty circles outside the cladogram denote the species in which the BGC/core gene was not detected by BiG-SCAPE but rather by the local sequence similarity search using the sequence of the core gene (similarity threshold > 80%). A total of 75.67% of the species (84 species in total) contained two SQS clusters, one belonging to each clan. The core gene in the BGCs of both clans is a putative SQS, but the two clans contain different accessory genes. The Dothideomycete and Eurotiomycete SQS BGCs, however, were phylogenetically most distant and shared low conservation with those of lichenized fungi belonging to the class Lecanoromycetes. Based on this evidence, we propose that Clan2 might be restricted to lichenized fungi. However, a broader sampling is required to confirm this observation. On the other hand, Clan1 is conserved in LFF but also shared by some closely related non-lichenized fungi

The combined results, i.e., the presence/absence and distribution of SQS BGCs of both clans across LFF as detected by BiG-SCAPE and based on local BLAST, were visualized using iTOL (Fig. 2).

### Core terpene BGCs of lichens – gene sequence conservation and BGC synteny

**Fig. 3:**
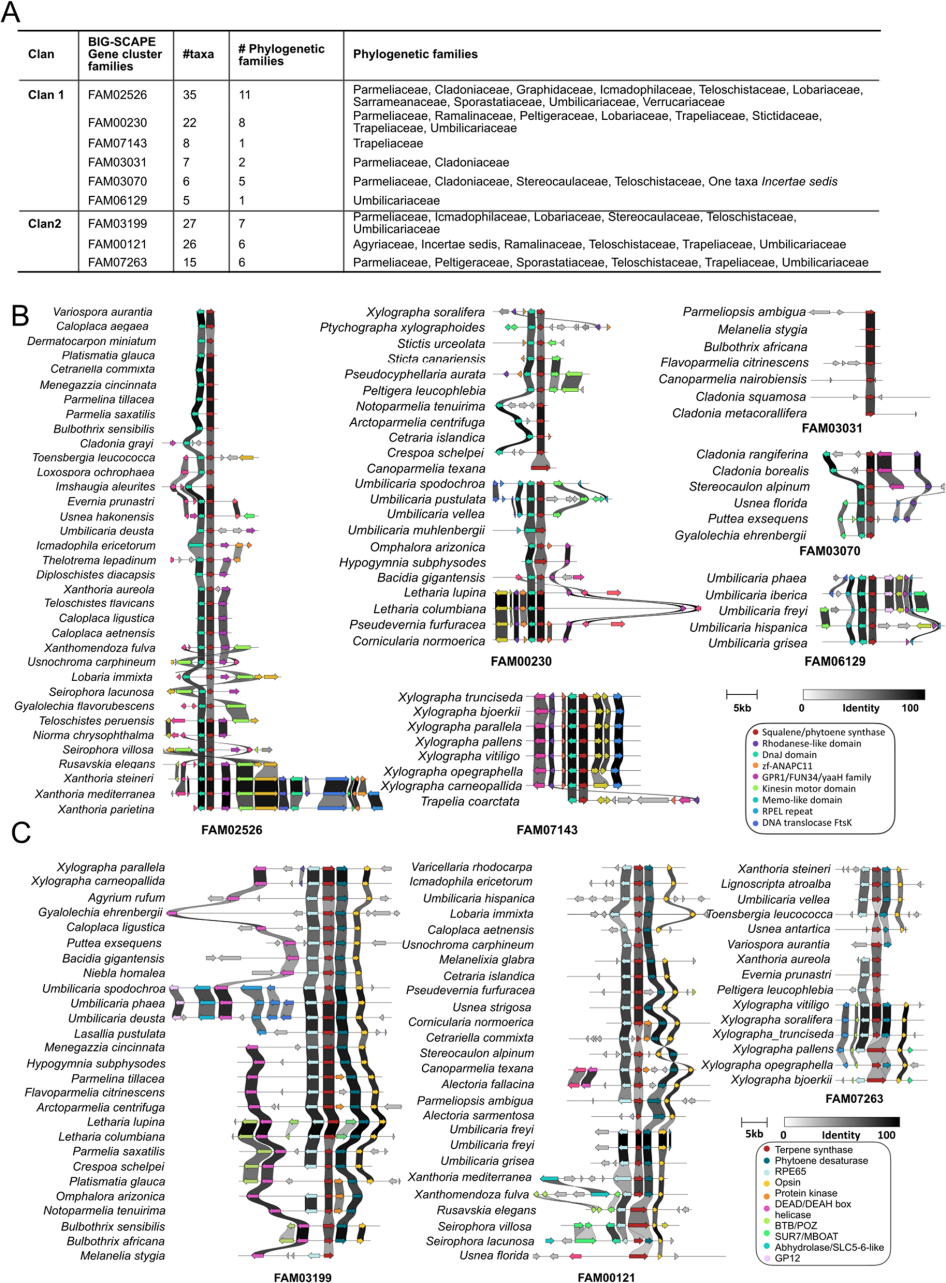
The two widely distributed putative SQS clans in the terpene network inferred by BiG-SCAPE at a raw-distance cutoff of 0.6 (Clan1 and Clan2 in Fig. 1B). **A** Table giving an overview of the gene cluster families constituting the two clans, including the number of species in which the BGC is present and their taxonomic distributions. Each clan is composed of a few gene cluster families (GCFs); Clan1 comprises six GCFs (FAM02526, FAM000230, FAM03031, FAM07143, FAM03070 and FAM06129), and Clan2 comprises three GCFs (FAM03199, FAM00121 and FAM07273). Each GCF contains several homologous terpene BGCs. **B** Clinker plot showing the synteny of squalene/phytoene BGCs in the six GCFs belonging to Clan1. The colored bands between the genes of the BGC indicate sequence similarity between genes. Notably, the position of accessory genes is not conserved among species, and certain genes, such as DNAJ proteins, may be present immediately upstream of SQS or after a few genes. Similarly, the kinesin motor domain (represented by the florescent green arrow in FAM02526) can be present upstream or downstream of the SQS. **C** Clinker plot showing the synteny among SQS BGCs present in the GCFs belonging to Clan2. As in Clan1, gene organization and location are variable among species. Four genes—RPE65, SQS (core gene of the BGC), phytoene desaturase and opsin—are common among the three GCFs of Clan2. While gene arrangement in most cases follows the above-mentioned order, there are some interesting exceptions. For instance, in some cases, protein kinase is present between squalene synthase and phytoene desaturase. Similarly, although the DEAD helicase is conserved and present in most species, its position varies among species. In rare cases, genes such as protein kinases are sporadically present between SQS and phytoene desaturase. This indicates that gene loss/gene gains are common in the evolution of SQS BGCs

The synteny and homology of the genes of each of the two widely distributed BGCs among species were displayed using Clinker, a pipeline to visualize BGC comparisons [54]. Collinearity analysis was performed between the members of Clan1 and Clan2, and the resulting figure was adjusted for presentation using Inkscape (Fig. 3). The core gene of both clans was identified as a putative SQS, based on the similarity to characterized biosynthetic genes in the MIBiG repository. However, most of the accessory genes could not be identified. To identify the accessory genes of the clans, we performed a BLASTp search on the individual genes of the cluster and a conserved domain search on the amino acid sequences of the genes upstream and downstream of the SQS gene.

We were then interested in assessing whether the two widely distributed SQS BGCs in LFF were specific to LFF or also shared by non-lichenized fungi. To test this hypothesis, we performed network construction on an extended dataset that included non-lichenized fungi belonging to Eurotiomycetes and Dothideomycetes and lichenized fungi belonging to Lichinomycetes (Supplementary Material 1, extended dataset).

### Terpene synthase classification and motif/domain annotation

The putative SQS BGCs from LFF did not group with the corresponding cluster from *Aspergillus* in the terpene network. This suggests that lichen SQS clusters are evolutionarily different than that of *Aspergillus* and non-lichenized fungi in general. We would like to mention that MIBiG provides the sequence similarity of individual genes, whereas BiG-SCAPE deduces BGC synteny and similarity. As the putative SQS did not group with its fungal or plant counterpart in MIBiG, we used the following indices to validate that the terpene synthase in both clans was indeed an SQS: sequence similarity, BGC synteny, presence of conserved motifs, and other physiochemical features (hydrophobicity and polarity of the domains).

We first tested the presence of characteristic terpene synthase and SQS domains in our sequences. Bacterial, fungal, and plant terpene synthases have characteristic, conserved metal-binding domains, namely (D/N)DXX(D/E) or DDXXXE [16]. In bacteria and fungi, this motif is located within 80–120 aa of the N-terminus. In addition, there is a 30 aa region at the C-terminus specific to fungal SQSs. We tested the presence of these regions in putative LFF SQSs by aligning the putative SQS sequences of LFF (Clan1 – FAM02526, FAM00230, FAM03031, FAM03070, FAM6129 and FAM07143) to those of non-lichenized fungi (*Aspergillus flavus*, *A. niger*, and *Candida albicans*) and a protozoan SQS from *Trypanosoma cruzi*. The sequences were aligned using GENEIOUS v.5.4 [55] implementing the standard MUSCLE algorithm, and gaps were treated as missing data. The purpose of this analysis was 1) to identify the characteristic conserved metal-binding motifs [(D/N)DXX(D/E) or DDXXXE] reportedly located within 80–120 aa (bacterial and fungal terpene synthases), 2) to confirm whether the C-terminal region, ~ 30 aa long, previously reported from *Aspergillus* (Eurotiomycetes, Ascomycota) to be fungal specific, is indeed fungal-specific and widespread in fungi by testing its presence in Lecanoromycete, Dothideomycete and Lichinomycete fungi, and 3) to identify LFF-specific amino acids in these domains, if any.

Another evidence to support that the widely distributed BGCs in Lecanoromycetes is an SQS is whether the physiochemical properties of the encoded protein align with those of a transmembrane protein. To test whether the biosynthetically identified putative SQS from LFF is indeed a transmembrane protein and to predict the transmembrane, intracellular and extracellular regions of SQS, we implemented two programs, Phyre2 [56] and DeepTMHMM [57] (https://dtu.biolib.com/DeepTMHMM). TMHMM is a bioinformatics tool based on the hidden Markov model (HMM) used to predict transmembrane helices of proteins. DeepTMHMM is a deep learning TMHMM model-based algorithm that predicts the protein structure and membrane topology of both alpha-helical and beta-barrel transmembrane proteins using deep learning and computes the probability of whether the protein is situated inside or outside of the cell and whether it is a transmembrane protein (comprising a high percentage of hydrophobic amino acid residues). Phyre2, on the other hand, predicts the three-dimensional (3D) structure of the protein from the given amino acids using the alignment of hidden Markov models. We then checked the physiochemical properties (i.e., hydrophobicity, etc.) of the entire sequence, as we identified the region predicted to be transmembrane (22 aa) using HeliQuest (http://heliquest.ipmc.cnrs.fr) [58]. This program returns hydrophobicity ⟨H⟩ and hydrophobic moment ⟨μH⟩ values for the input sequences. A positive ⟨H⟩ value (e.g., > 1) denotes a hydrophobic helix, and a negative value indicates a hydrophilic helix.

### Gene tree estimation for Clan1 and Clan2 squalene synthases

In addition, to test the sequence conservation among the putative SQSs of the two clans and of the SQSs of these clans with those of non-lichenized fungi (*Aspergillus* sp., *Candida albicans*), and the flagellated protozoan species, *Trypanosoma cruzi*, we aligned the abovementioned sequences using GENEIOUS v.5.4 [55], with the standard MUSCLE algorithm [59], and treated gaps as missing data. This alignment was then used to infer a 1000 bootstrap maximum likelihood tree [47, 60, 61] to test the evolutionary distance between these SQSs found in LFF and of the LFF SQSs to those of the model taxa mentioned above (Additional Supplementary Material 3 showing species tree constructed from single copy BUSCOs of 111 lichens).

## Results

### BUSCO completeness

Total 111 LFF genomes were included in the study, including 22 de novo sequenced ones. The dataset comprises 102 Lecanoromycetes, four Eurotiomycetes and five Dothideomycetes LFF (Supplementary Material 1). For the newly sequenced genomes, raw reads as well as genome assemblies are available at NCBI (accessions SAMN41602368- SAMN41602389, represented in bold in the Supplementary Material 1). BUSCO was run to estimate the genome completeness and to obtain the single copy orthologs for species tree inference. The species tree was then inferred from the single copy BUSCO orthologs. The BUSCO completeness estimated using the Ascomycota database is  mentioned in Supplementary Material 1. Seventy six percent of the genomes had a high completeness, i.e. > 90%. For the genomes with a completeness lower than 90% the absence of SQS was validated in the raw data.

### Biosynthetic gene diversity and BGC networks

Biosynthetic genes were predicted in all 111 genomes using the AntiSMASH; this unraveled the presence of total 5,542 regions that contain BGCs. The majority of the BGCs belong to the class polyketide synthases (PKSs), non-ribosomal peptide synthases (NRPSs), terpenes and RiPPs (ribosomally synthesized post-translationally modified peptides) (1,939, 1,278, 724 and 883 BGCs, respectively). Note that the absolute number of regions identified by AntiSMASH and BGCs may not match as certain regions contain more than one BGC. BiG-SCAPE analysis generated network for each BGC class. Within each network, similar BGCs were grouped into GCFs, and similar GCFs were further organized into clans. BGCs without any similar biosynthetic genes within the dataset or in the MIBiG repository appeared as singletons in the network. Although several clans were obtained for each BGC network—PKSs, NRPSs, RiPPs, hybrids and terpenes—most of them comprised only a few  BGCs. The largest clans, comprising more than 80 BGCs, were obtained only for terpene network, thereby making the constituent BGCs the most promising candidates for being conserved across LFF.

### Terpene BGCs: diversity and similarity network

Among the BGCs identified by AntiSMASH, 724 (13%) putative terpene BGCs were predicted. Among these, 680 terpene BGCs were detected in Lecanoromycetes (6.6 ± 3.2 BGCs/taxon), 24 in Eurotiomycetes (6.75 ± 1.2 BGCS/taxon) and 29 in Dothideomycetes (7.4 ± 2.07 BGCS/taxon) (Fig. 1A). Physciaceae constituted the most terpene BGC-rich family (14.5 ± 2.1 BGCs/taxon) (Fig. 1A, Supplementary Material 1). The most terpene BGC-rich taxa were *Parmelia* spp. (12.5 + 3.5 BGCs/taxon), *Bulbothrix* spp*.* (13.5 + 0.5 BGC/taxon) and *Evernia prunastri* (14 BGCs/taxon). None of the terpene BGCs detected in the dataset grouped with a previously characterized terpene BGC present in the MIBiG repository. This indicates that LFF terpene BGCs are putatively structurally and functionally novel. This could be because MIBiG mostly comprises plant terpene BGCs, which are diterpenes, whereas LFF are known to produce triterpenes; therefore, the BGCs reported here may thus be involved in triterpene synthesis.

BiG-SCAPE program, used to cluster the similar biosynthetic genes, demonstrated the presence of both species-specific and highly conserved terpene BGCs in LFF. The terpene network comprised 724 terpene BGCs, which were grouped into 445 GCFs based on the sequence similarity of core genes and BGC synteny, thus highlighting the high diversity of the LFF terpene synthases (Fig. 1B). Of these, about 51% (377) were singletons (detected only once and unique to a taxon), whereas others were grouped into GCFs with two or more  BGCs. Most GCFs were small and comprised only the terpene BGCs from a few species (e.g., 418 terpene GCFs out of 445—94%—were composed of three or fewer BGCs).

Interestingly, we found two widely distributed BGCs, represented by Clan1 and Clan2 in the terpene network (Fig. 1B). Clan1 consisted of six GCFs and 83 BGCs, and Clan2 consisted of three GCFs and 68 BGCs (Fig. 3A). As only one copy per clan was present in an organism, Clan1 and Clan2 BGCs were present in 83 and 68 species respectively. Clan1 was present in  94.6% of the species encompassing all the families included in the study (missing in six species, Fig. 2) whereas Clan2 was shared across 80% of the species (missing in 23 species, Fig. 2).

### Core terpene BGCs of lichens

Both widely distributed terpene clans have a putative SQS as the core gene in the member BGCs. Apart from these two widely distributed clans, two other clans, Clan3 and Clan4, also contain a putative SQS. These clans were composed of five and four BGCs and were detected via local BLAST. Clan3 comprised LFF belonging to Dothideomycetes whereas Clan4 comprised putative SQS BGCs from Eurotiomycetes. The segregation of Clan3 and Clan4 BGCs as independent GCFs despite the presence of SQS indicates that the sequence and architecture of these BGCs is divergent from those of Clan1 and Clan2 (Fig. 1B).

Overall, 75% of the LFF contained two copies of SQS, one each belonging to Clan1 and Clan2 (Fig. 2). Each taxon had at least one copy of the SQS. Interestingly, the gene composition of the SQS cluster of Clan4 (comprising only Eurotiomycetes LFF) is somewhat similar to that of Clan1 (composed of Lecanoromycete LFF), as they both contain the same accessory gene (i.e. a gene containing the conserved DnaJ domain). However, unlike in Clan1, where this gene is present immediately upstream of SQS, in Clan3, it is present downstream (Fig. 3A and Supplementary Material 2). The SQS gene is divergent among clans, as depicted by its phylogenetic relationship and distance matrix (Supplementary Materials 3 & 4).

### Evolutionary conservation of SQS BGCs: Clan2—predominantly found in LFF; Clan1—widely distributed in LFF but also present in closely related non-lichenized *fungi*

This study primarily focused on detecting widely distributed, conserved BGCs in LFF, mainly within Lecanoromycetes. To further assess whether the BGC was restricted to LFF, we included LFF belonging to Lichinomycetes and non-lichenized fungi from the closely related classes Eurotiomycetes and Dothideomycetes (Supplementary Material 5). The Clan2 SQS BGC was widely distributed in the Lecanoromycete LFF but was not detected in non-lichenized fungi belonging to Dothideomycetes and Eurotiomycetes (see Supplementary Material 5). A BLAST search confirmed the presence of the corresponding SQS in Eurotiomycete and Dothideomycete LFF but it was not detected in the non-lichenized fungi belonging to these classes.

In contrast, the Clan1 SQS BGC was present not only in lichenized fungi from all three classes (Lecanoromycetes, Eurotiomycetes and Dothideomycetes) but also in non-lichenized parasitic fungi belonging to the Dothideomycetes (Supplementary Material 5, Additional Supplementary Material 2, showing Clan1, Clan2, SQS-BGC trees). Based on this evidence, we propose that Clan2 is predominantly found in LFF, whereas Clan1 is widely distributed in LFF but also shared by some closely related non-lichenized fungi.

### Gene cluster composition of conserved clans

Clan1 BGCs contain two genes conserved across LFF, an SQS and a J domain-containing protein. In contrast, some genes in Clan1 BGCs are conserved only within a fungal family or genus, e.g., abhydrolase/SLC5-6-like and BTB/POZ domain-containing proteins (based on BiG-SCAPE domain identification) (Fig. 3A, B). The BTB/POZ domain (broad-complex, tramtrack, and Bric à brac or poxvirus and zinc finger) is an evolutionarily conserved protein–protein interaction domain mainly found in transcription factors. The J domain-containing protein is located upstream of the SQS. These accessory genes are usually involved in protein (re)folding, trafficking, remodeling, disaggregation, and degradation. Other proteins frequently found in the conserved BGCs of Clan1 have conserved domains typically found in proteins related to transport and signaling functions, such as protein kinases (cellular signaling), DNA translocase FtsK (filament temperature sensitive mutant K) and abhydrolase/SLC5-6-like (localization and transport) and transcription regulation (the BTB/POZ domain containing protein).

Clan2 BGCs contain four genes that are conserved across its member species (i.e., present in 90% of the BGCs), namely, SQS, phytoene desaturase, RPE65 and opsin (Fig. 3C). RPE65 is most likely involved in regulatory functions and is located upstream of SQS, whereas phytoene desaturase and opsin are located downstream of SQS. Other genes occasionally present in the clan include protein kinase, abhydrolase, GPI2, helicase, etc. The core gene of Clan2 was slightly shorter than that of Clan1 (Clan1: 1000–1600 bp, 330–550 aa vs. Clan2: 900 bp, 300 aa long) (Fig. 3B, C). While the SQSs of the Clan2 BGCs displayed high sequence similarity among them, both the gene sequence and content differed from those of the Clan1 BGCs (Fig. 3B, C).

### Terpene synthase identification and motif/domain annotation

To assess if the widely distributed BGCs in Lecanoromycetes have an SQS as the core terpene synthase, we estimated the sequence conservation and physiochemical properties of the encoded protein. We found that the conserved, characteristic metal-binding motif of terpene synthases (D/N)DXX(D/E) was present in the BGCs of both conserved clans across LFF in the same region as in bacterial, fungal, and plant terpene synthases, i.e., within 80–120 aa from the N-terminus. We identified this region as DT(I/V)EDD in our dataset, and the location of this region is also similar to what is known from bacteria, fungi, and plants, i.e., 50–100 aa from the N-terminus. This region includes protein‒protein interaction motifs and has been previously suggested to be specific to fungi (based on evidence from non-lichenized fungi [62]). As expected, this region was similar and conserved between the lichenized and non-lichenized fungi in the dataset. Interestingly, we found the C-terminus region to be highly variable between LFF and non-lichenized fungi.

**Fig. 4:**
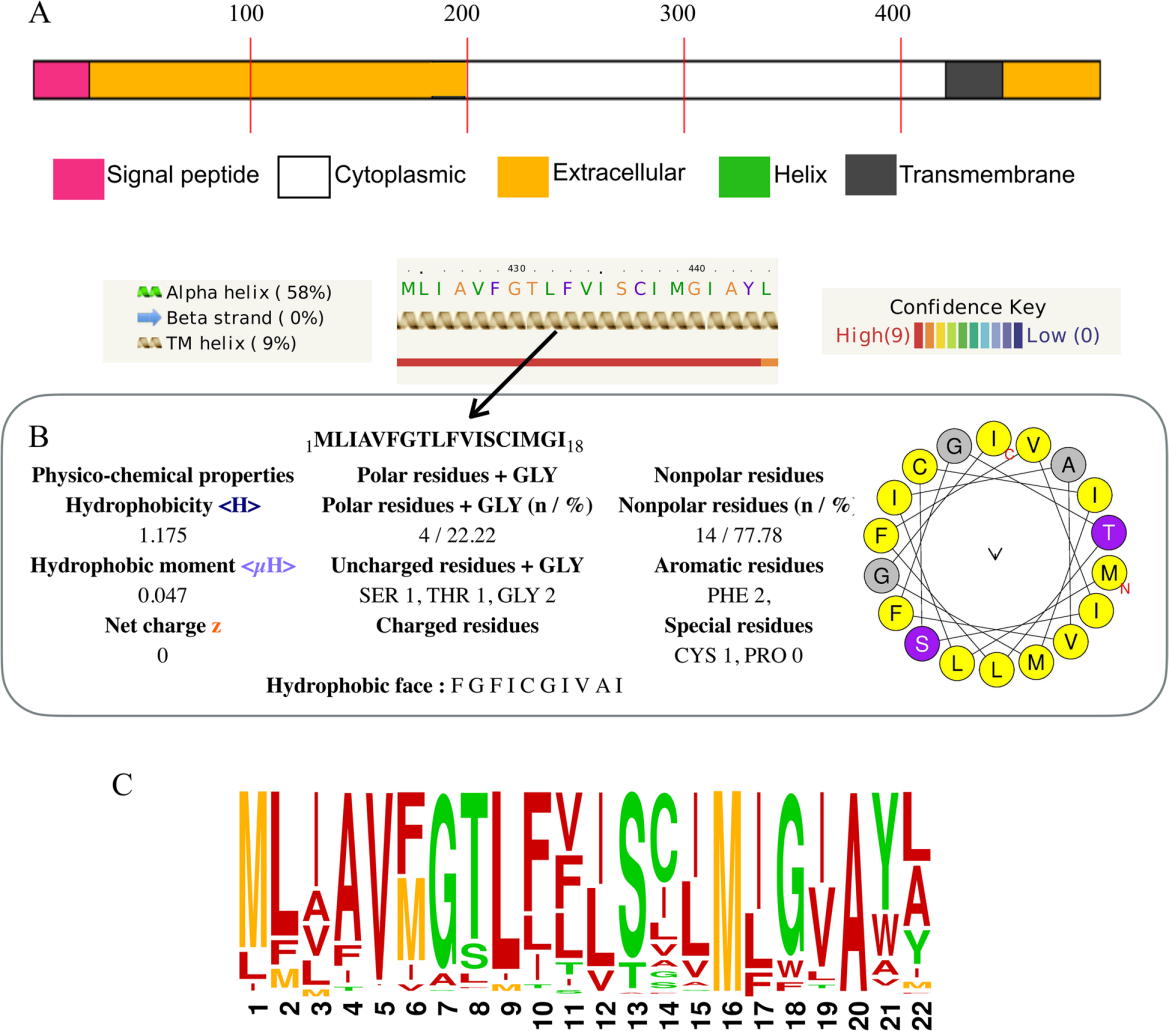
**A** The domains and motifs of LFF squalene synthase derived from the consensus sequence as predicted by DeepTMHMM. The program detected a signal peptide, extracellular and cytoplasmic regions at the N-terminus and a transmembrane region at the C-terminus. **B** The high hydrophobicity of the predicted transmembrane region, as estimated by HELIQUEST, further supports that the region is transmembrane. HELIQUEST calculates the physicochemical properties (hydrophobicity and percentage of polar versus nonpolar residues) and amino acid composition of an α-helix and scans a databank of proteins to identify protein segments with similar features in each protein sequence. **C** Sequence logo created using WebLogo (https://weblogo.berkeley.edu/logo.cgi [63]) depicting the conservation of the fungal-specific region of the squalene synthases of Clan1 across Lecanoromycetes

We additionally checked for the presence of highly hydrophobic residues toward the C-terminus that form the transmembrane domain of the protein, which is one of the typical features of SQSs. DeepTMHMM predicted the presence of one transmembrane helix, whereas Phyre2 also detected an additional helix toward the N-terminus. However, the second transmembrane helix identified by Phyre2 had a low probability of being a transmembrane region. We propose that the putative squalene synthase of LFF has only one transmembrane helix of approximately 22–25 aa present towards the C-terminus (Supplementary Material 6). The protein contains intracellular and extracellular regions of approximately 420–430 bp and 40–50 aa before and after the transmembrane helix, respectively (Fig. 4A, B). Furthermore, the putative SQS has a C-terminal region that is typical of fungal SQSs and is absent in plants, fungi and humans. In fact, *Aspergillus fumigatus* SQS contains a conserved hinge region of 26 aa before the transmembrane domain, as detected in LFF (Supplementary Material 6). We also identified this region in LFF and found it to be highly conserved across the class, except for a few variations (Fig. 4C, Supplementary Material 6).

Fungal SQSs are transmembrane proteins with polar, intra- and extracellular regions and a hydrophobic transmembrane helix. The predicted transmembrane region present towards the C-terminal region of LFF SQS has similar properties (a high percentage of polar residues, high hydrophobicity, and hydrophobic moment (Fig. 4A, B)) to that of the C-terminal helical domain of *Aspergillus fumigatus* squalene epoxidase, suggesting that it synthesizes a transmembrane protein as reported in *Aspergillus fumigatus*. The membrane-spanning portion of the SQS is also highly variable within LFF, as was shown within each kingdom of life. This region has the physiochemical properties typical of a transmembrane region (high hydrophobicity), while having low sequence similarity. The TM domain of LFF SQS is slightly more polar (22% polarity) than that of *Aspergillus flavus* and human SQSs (11% and 16% polar residues, respectively, in the TM domain) but less polar than that of *Trypanosoma cruzi* (33% polar residues). As the SQS transmembrane domain potentially anchors the SQS to the endoplasmic reticulum, the difference in the polarity of the TM domain may indicate a somewhat different binding potential.

Before the fungal-specific C domain there is a hinge sequence of 26 aa linking the catalytic and transmembrane domains, which is highly conserved within each kingdom of life [62]. We found this region to be highly conserved in LFF as well.

### Gene tree estimation for LFF SQSs

The final alignment generated by aligning 160 SQS sequences comprised 672 aa. In the phylogenetic tree displaying the evolutionary distance of all the SQS in the dataset (from all the species and both copies of the SQS as well as those of *Aspergillus* sp., *Candida albicans* and *Trypanosoma cruzi*) neither the SQS of Clan1 nor that of the Clan2 group with the *Aspergillus* sp., *Candida albicans* and *Trypanosoma cruzi* SQS. The Clan1 and Clan2 SQSs instead form independent clades. This indicates that the Lecanoromycete SQSs are divergent from those of non-lichenized fungi. Interestingly, in the SQS network, which was generated using an extended dataset that included non-lichenized fungi from Eurotiomycetes and Dothideomycetes, several non-lichenized fungal SQSs grouped within Clan1, demonstrating that despite being divergent from *Aspergillus* SQS (Eurotiomycetes), Clan1 SQS is shared by some distantly related, non-lichenized fungi belonging to Dothideomycetes. Clan1 SQS is present in both lichenized and non-lichenized fungi, present in Lecanoromycetes, Eurotiomycetes and Dothideomycetes. In contrast, Clan2 comprises only LFF SQSs, including LFF belonging to Lichinomycetes, Dothideomycetes and Eurotiomycetes (Supplementary Material 5).

## Discussion

In this study, we explore the diversity of biosynthetic gene clusters associated with terpene biosynthesis in lichen-forming fungi. We also investigate whether any of these BGCs are evolutionarily conserved and examine the evolutionary forces that influence the organization of these BGCs in lichen-forming fungi.

### Taxonomic distribution of BGCs linked to terpene biosynthesis

Our study showed that terpene BGCs are widespread among LFF, and all lichenized fungi have at least one BGC for synthesizing terpenes (Fig. 1A). Although several structurally diverse and unique triterpenes have been reported from LFF [64], this is the first study reporting terpene-related biosynthetic gene clusters. Interestingly, except for the two terpene BGCs that were widespread among LFF, most were singletons bearing no similarity to other terpenes in lichens or to any other terpenes identified thus far. This indicates that LFF terpene BGCs are mostly lineage-specific (Fig. 1B). This is also the case for plant terpene-related BGCs, some of which are widely distributed (e.g., cholesterol synthase), while others have a more restricted, taxon-specific distribution (e.g., carotenoids, vitamins A and D, steroid hormones, pheromones, essential oils such as camphor and defense-related metabolites; [65, 66]). The presence of rare, species- or lineage-specific terpenes in non-lichenized fungi has also been reported. For instance, *Aspergillus ochraceus* produces several sesquiterpenoids which are rare [67]. Similarly, the marine fungus *Eutypella scoparia* produces a 7-methyl oxidized 2-carene derivative [68], and the marine fungus *Cochliobolus lunatus* produces two dendryphiellins, both of which are rare terpenes [20]. The presence of numerous taxon-specific terpenes reflects the breadth of the functional potential and species-specific function of these genes in fungi. Given the wide range of ecological niches of lichens, it is probable that lineage-specific terpenes play specific roles in lichen biology, such as biotic and abiotic stress responses and defense.

### Are there conserved BGCs in lichens?

Two BGCs are widely distributed across LFF (members of Clan1 and Clan2 in Figs. 1B and 2). Both BGCs have an evolutionarily stable SQS as the core gene and a set of dynamic lineage-specific accessory genes (Fig. 3B, C). It is to be noted that Clan1 BGCs are homologous to each other but not to those of Clan2 BGCs and vice versa. Seventy-five percent of LFF have two copies of SQS, one from each clan. Each taxon has at least one copy of the SQS, showing 100% conservation in LFF (Fig. 2). In fact, SQS is conserved across kingdoms and has been reported in fungi, plants and animals, including rats and humans [62, 69]. However, BGC organization differs among them, generating diverse sterols among organisms [62, 69]. For instance, the SQS BGC encodes ergosterol in non-lichenized fungi, ß-carotenoids in plants and cholesterol in mammals/humans [70]. Squalene synthase is one of the starting enzymes in the sterol pathway (catalyzes the first step in sterol biosynthesis – condensation of two molecules of farnesyl diphosphate (FPP) to form squalene, the precursor of all steroids), and consecutive enzymes dictate the final protein produced [71, 72]. Ergosterol, for instance, differs from the predominant mammalian sterol, cholesterol for the presence of a methyl group, among other differences, and hence requires methyl transferases [72, 73].

The LFF SQS gene shows low sequence similarity to the corresponding genes between clans and from non-lichenized fungi (Supplementary Materials 3 and 4). Furthermore, the gene composition of the two SQS BGCs is also different. This widespread presence of SQS BGCs and high SQS sequence divergence, coupled with the presence of different accessory enzymes in the cluster, suggest that the two SQS BGCs might be involved in important, slightly different metabolic pathways putatively related to sterol biosynthesis.

Of the two conserved SQS BGCs, those belonging to Clan2 are composed of LFF SQSs belonging to Lecanoromycetes, Dothideomycetes, Eurotiomycetes and Lichinomycetes, whereas those of Clan1 contains SQSs from both LFF and non-lichenized fungi, including parasitic fungi belonging to Dothideomycetes (Fig. 3A, Supplementary Material 5). Based on this observation, we propose that both SQS BGCs are widely distributed in LFF but those belonging to Clan2 are specific to LFF. On the other hand, Clan1, in addition to being present in LFF, is also shared by parasitic non-lichenized fungi.

The presence of an evolutionarily conserved BGC indicates the functional relevance of the BGC for the organism [74–76]. Non-lichenized fungi produce ergosterol via a complex process involving several enzymes, such as oxidases, methyltransferases, demethylases, desaturases and isomerases. Studies suggest that some lichens produce ergosterol [77–80] but neither of the conserved SQS BGCs we found have the same BGC composition as that reported for the *Aspergillus* ergosterol cluster [71, 81], implying that lichens either produce a slightly different variant of ergosterol or that enzymes outside the BGC may participate in ergosterol synthesis. Clan2 BGCs contain desaturase and oxidases required for ergosterol synthesis (Fig. 3C) but these genes are not the same as those reported for *Aspergillus* [71, 81]. Ergosterol or sterols are involved in cell wall maintenance and the regulation of membrane fluidity and structure [73]. Given the symbiotic nature of LFF, it is expected that the membrane structural requirements to sustain fungal-algal cross-talk are different from those of non-lichenized fungi, which may explain the difference in BGC architecture between the two.

Our study overturns the notion that secondary metabolite BGCs, unlike primary metabolite BGCs, show a narrow taxonomic distribution. The presence of an evolutionarily conserved biosynthetic pathway in LFF is certainly intriguing, considering that until now, the lichen BGCs were only reported to have a narrow, almost lineage specific, taxonomic distribution [4, 7–9, 11, 13, 15, 82].

### Gene duplication and squalene synthase isoforms

The presence of two SQS isoforms in LFF suggests a possible gene duplication of this gene, followed by an independent evolutionary pathway leading to different BGC architectures and, most likely, different functions. Copy number variation for SQS BGC is a common phenomenon and is an important factor in the evolution of these genes, especially in plants. For instance, a single copy of this gene has been reported in rice [83], Japanese yew *Taxus cuspidate* [84] and the petroleum plant *Euphorbia tirucalli* [85], whereas two copies have been reported from tobacco [86], Russian dandelion *Taraxacum koksaghyz* [87], the liquorice plant *Glycyrrhiza glabra* [88], the barrel medic plant *Medicago truncatula* [89] and *Arabidopsis thaliana* [90]. In rare cases, more than two copies have been found, for instance three copies in the ginseng plant *Panax ginseng* [91].

### Squalene synthase BGC in lichen-forming *fungi*

We found that both the conserved BGCs contain putative SQSs as the core gene even though the accessory genes are different among LFF SQS BGCs as well as from the SQS BGC in non-lichenized fungi (Fig. 3B, C). In Clan2, in addition to SQSs, carotenoid biosynthesis enzymes—phytoene desaturase, RPE65 and opsin—are highly conserved in LFF. Phytoene desaturase is present immediately downstream of putative SQSs (Fig. 3B). Studies have shown that desaturases desaturate squalene to make dehydrosqualene and subsequently carotenoid pigments. Opsin, which is present further downstream of phytoene desaturase, is also highly conserved in these BGCs. Opsins are universal photoreceptors. It has been proposed that they are involved in the production of carotenoid molecules with photoprotective or antioxidant effects by acquiring promiscuous desaturases [92].

The accessory genes in the conserved SQS BGCs differed from those present in the SQS BGC of the non-lichenized fungi. For instance, the SQS BGCs of fungi contains oxidative enzymes of the cytochrome P450 monooxygenase (CYP450) family, which are involved in functional modifications and hence diversification of terpenes [66, 93, 94]. Interestingly, we did not find any cytochrome family genes in the putative SQS BGC in LFF, instead a gene putatively a RPE65 family protein (retinal pigment epithelium-specific 65 kDa protein)—a retinal gene belonging to the carotenoid oxygenase family protein. Interesting this gene, like CYP450 also codes for an enzyme involved in the oxidation of its substrate. However, without experimental validation it is impossible to establish if RPE65 performs similar function as CYP450 in SQS cluster. Our study suggests that the sterol pathway in LFF may involve genes other than those reported for non-lichenized fungi.

### Evolutionary forces shaping the composition of SQS BGCs in LFF

Although the two SQS BGCs are mostly conserved in LFF, they differ in gene content and organization among them. Some genes have a generally conserved pattern of organization whereas, some others are dynamic with regard to their location in the SQS BGC. For instance, in Clan1, the order of Rhodanese-like domain containing protein synthase and SQS is mostly conserved (Fig. 3B). Similarly, in Clan2, the gene order for RPE65, SQS, phytoene desaturase and opsin is mostly the same (Fig. 3C). In general, gene order in prokaryotic as well as eukaryotic genomes tends to be poorly conserved throughout evolution [95–97]. However, certain groups of genes remain adjacent to each other in the genome even over long evolutionary distances, which suggests that selection tends to preserve their genomic colocalization [98]. The preservation of gene order indicates functional relationships, as highly conserved gene pairs are far more likely to be functionally related than those that are poorly conserved.

On the other hand, certain genes in the SQS BGCs in LFF have species- and genus-specific gains/losses and structural rearrangements. For instance, helicase genes in these BGCs are present in only two species. Similarly, a putative alpha/beta hydrolase gene is present in only two species and shows location and orientation differences between them (Fam121 in Clan2, Fig. 3C). Another example is the gene coding for protein kinases, present only in some species among the members of Clan2 (Fig. 3C). Interestingly, this gene is found in some species of the family Parmeliaceae between the SQS and the phytoene desaturase genes, disrupting the otherwise conserved arrangement of these two genes adjacent to each other. The sporadic presence of this gene in certain species indicates that gene gain/loss is an essential process in the evolution of the SQS BGC in Parmeliaceae. Events such as gene duplications/losses have been associated with functional divergence of BGCs. For example, gene rearrangements in trichothecene BGCs are linked to the diversity of trichothecene toxins in plant pathogenic fungi [99]. Similarly, sterigmatocystin and aflatoxin BGCs are evolutionarily related but differ in gene content, order and orientation, with aflatoxin producers having extra genes that facilitate the downstream steps for the conversion of sterigmatocystin into different types of aflatoxins [100]. The accessory gene family combinations in the BGC further potentially increase the functional plasticity of these genes, tailoring the product to an organism’s specific needs. The different accessory genes and their organization in the BGC could be related to slightly different species-specific modifications of the final product in Lecanoromycetes.

### Sequence conservation among LFF SQSs

The putative SQSs in LFF contain several conserved regions. In particular, we found hinge region to be highly conserved . This region is present before the fungal-specific C domain and links the catalytic and transmembrane domain regions. This regions, as observed in other kingdoms of life, displays high conservation among taxa [62]. In non-lichenized fungi, it is involved in the assembly of ergosterol multienzyme complexes [71, 73].

Additionally, for the first time, we report the fungal specific C-terminal region of the LFF SQSs (Supplementary Material 6). This region is absent in plants, bacteria and humans. Previously, a small stretch of amino acids at the C-terminal region was reported to be fungal specific based on comparative studies on model fungi from Eurotiomycetes (*Aspergillus* spp.) and Saccharomycetes (yeast) and other plant and vertebrate . Presence of this regions only in fungi is particularly interesting, and this property can be used to develop targeted antifungal and antiprotozoal therapeutics, i.e., kingdom-specific therapeutics [71, 101–103]. For instance, SQSs play a crucial role in the sterol pathway— involved in the cholesterol synthesis in mammalian cells and ergosterol in eukaryotic microorganisms such as fungi—essential for cellular membrane function and growth [72, 73, 104]. However, because certain regions of SQS are conserved across different kingdoms, therapeutics targeting fungal SQS face the challenge of potentially interfering with human SQS pathways. Antifungals targeting fungal-specific hinge regions offer a solution for preventing unwanted effects on human cells.

## Conclusions

We present the first in-depth analyses of terpene BGCs in LFF, including their distribution, diversity, and evolution. Our study provides new perspectives on the evolutionary conservation of these pathways. We show that all lichenized fungi have the potential to synthesize a variety of species-specific terpenes, which could be explored for their unique bioactivity. While most terpene BGCs are taxon specific, two are widely distributed in LFF. Interestingly, both widely distributed BGCs have a SQS as the core gene but different sets of accessory genes, indicating that gene duplication, loss and gain were the major evolutionary forces driving the evolution of these BGCs. However, additional studies are necessary to confirm these evolutionary events. Contrary to the previous belief that lichen metabolites are taxon-restricted, we revealed the presence of BGCs widely distributed in lichenized fungi for the first time. Furthermore, we provide the putative structure of the various domains in the squalene synthase gene, which has potential pharmaceutical implications. Our study sets a baseline for further exploration of terpene BGCs in lichens and its evolutionary significance.

## Supplementary Information

Additional file 1.

Additional file 2.

Additional file 3.

Additional file 4.

## Data Availability

The genomes used in the study are available at NCBI (accession numbers in the Supplementary Material 1). For the genomes generated for this study the accession numbers (SAMN41602368—SAMN41602389) are represented in bold in the Supplementary Material 1). The BiG-SCAPE results are provided as additional supplementary Material.
