# Supplementary material for: Are there conserved biosynthetic genes in lichens? Genome-wide assessment of terpene biosynthetic genes suggests ubiquitous distribution of the squalene synthase cluster
Source: BMC Genomics. 2024 Oct 7;25:936. doi: 10.1186/s12864-024-10806-0 (PMC11457338; doi:10.1186/s12864-024-10806-0)
Supplement: Supplementary file 1 — Additional file 1. [file 12864_2024_10806_MOESM1_ESM.zip › cutoff60_111taxa/cutoff60_newRIPPS-included_111taxa/index.html]

BiG-SCAPE v1.1.5


Biosynthetic Genes Similarity Clustering and Prospecting Engine  
Version 1.1.5

Networks:


Runs:

|  |  |  |
| --- | --- | --- |
|  |  |  |

Found BiG-SCAPE useful? Please consider citing our paper:
