# Supplementary material for: Are there conserved biosynthetic genes in lichens? Genome-wide assessment of terpene biosynthetic genes suggests ubiquitous distribution of the squalene synthase cluster
Source: BMC Genomics. 2024 Oct 7;25:936. doi: 10.1186/s12864-024-10806-0 (PMC11457338; doi:10.1186/s12864-024-10806-0)
Supplement: Supplementary file 4 — Additional file 4. [file 12864_2024_10806_MOESM4_ESM.zip › Supplementary Material/Supplementary_material5.pdf]

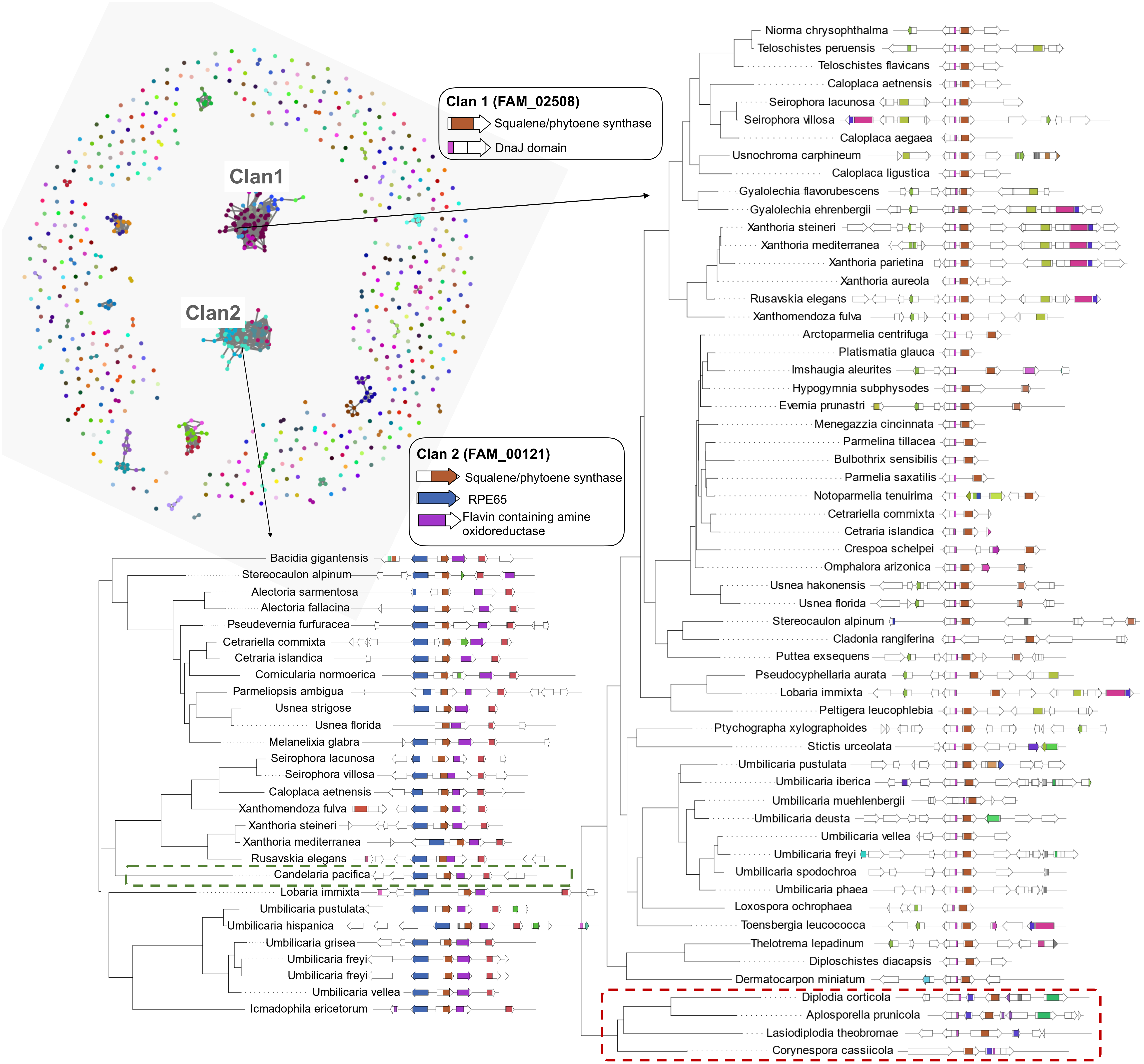

Supplementary material 4 : Network analyses of the test dataset. BiG-SCAPE clustering at threshold 0.6 using111 taxa from the main dataset and 10 non-lichenized Dothideomycetes fungi, eight non-lichenized Eurotiomycetes and four evolutionary distantly LFF belonging to Lichinomycetes. Clan2 comprises only of LFF, including one from Lichinomycetes (highlighted in green box). Clan 1 comprises mostly LFF but also a clade of parasitic fungi (highlighted by red box)
