# Supplementary figures and images for: Are there conserved biosynthetic genes in lichens? Genome-wide assessment of terpene biosynthetic genes suggests ubiquitous distribution of the squalene synthase cluster
Source: BMC Genomics. 2024 Oct 7;25:936. doi: 10.1186/s12864-024-10806-0 (PMC11457338; doi:10.1186/s12864-024-10806-0)

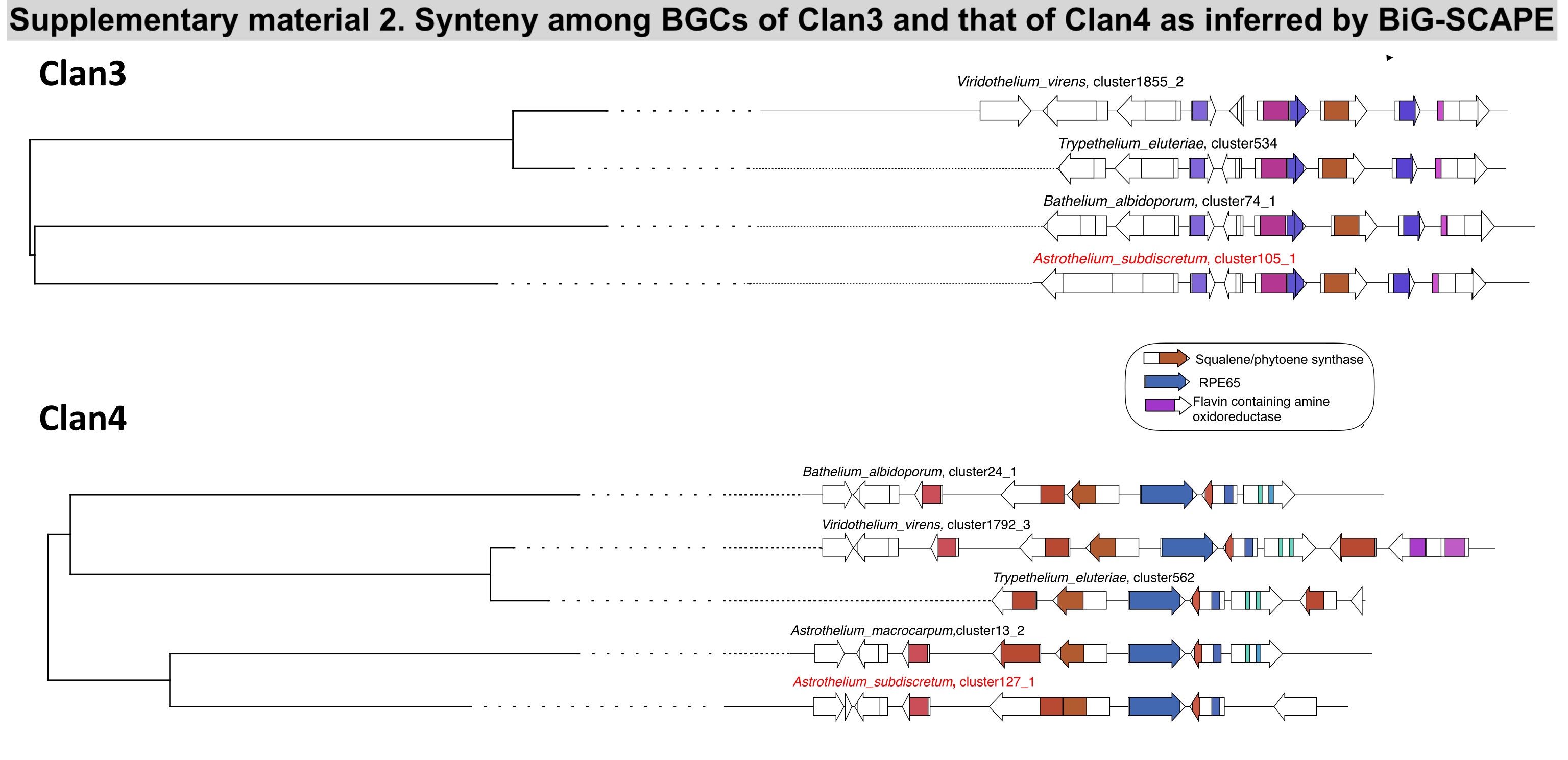

Supplement: Supplementary file 4 — Additional file 4. [file 12864_2024_10806_MOESM4_ESM.zip › Supplementary Material/Supplementary_material2_HQ.png]

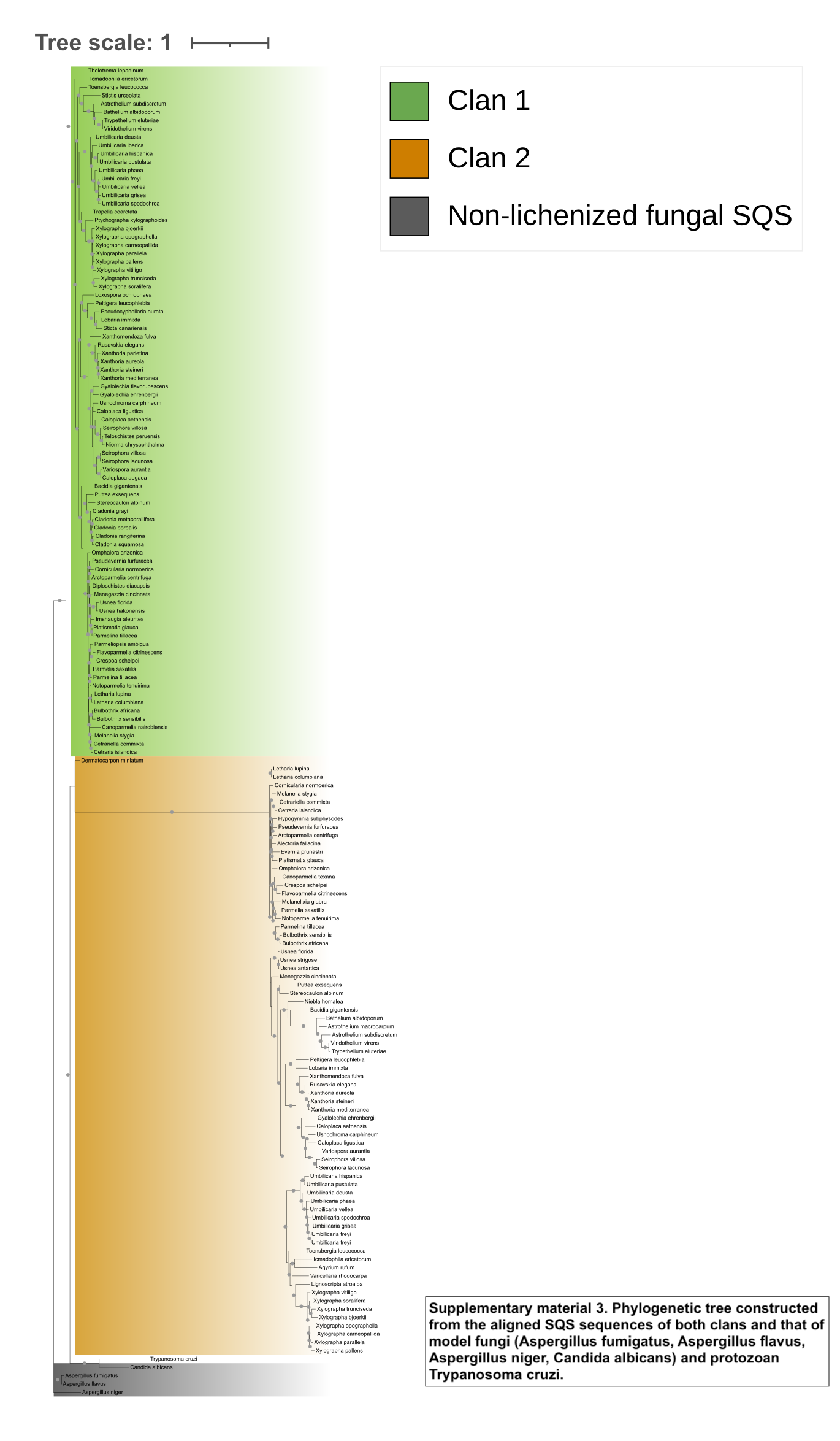

Supplement: Supplementary file 4 — Additional file 4. [file 12864_2024_10806_MOESM4_ESM.zip › Supplementary Material/Supplementary_material3.png]
