# Supplementary material for: Are there conserved biosynthetic genes in lichens? Genome-wide assessment of terpene biosynthetic genes suggests ubiquitous distribution of the squalene synthase cluster
Source: BMC Genomics. 2024 Oct 7;25:936. doi: 10.1186/s12864-024-10806-0 (PMC11457338; doi:10.1186/s12864-024-10806-0)
Supplement: Supplementary file 1 — Additional file 1. [file 12864_2024_10806_MOESM1_ESM.zip › cutoff60_111taxa/cutoff60_newRIPPS-included_111taxa/html_content/networks/2023-05-02_09-52-12_hybrids_glocal_lichens_c0.60/overview.html]

BiG-SCAPE Result


### Run Information

Analysis Started:

Parameters:

Analysis Completed:

### Input Data

Total Number of Genomes:

Total BGCs:

### Network Overview

Number of families:

Average number of BGCs per family:

Max number of BGCs in a family:

Families with MIBiG Reference BGCs:

GCF absence/presence heatmap

Cluster GCF based on:   


Show:   

20
50
100
All
largest GCFs

Cluster Genomes based on:

Download:
Absence/Presence table (tsv)

Loading data...
