# Supplementary material for: Are there conserved biosynthetic genes in lichens? Genome-wide assessment of terpene biosynthetic genes suggests ubiquitous distribution of the squalene synthase cluster
Source: BMC Genomics. 2024 Oct 7;25:936. doi: 10.1186/s12864-024-10806-0 (PMC11457338; doi:10.1186/s12864-024-10806-0)
Supplement: Supplementary file 1 — Additional file 1. [file 12864_2024_10806_MOESM1_ESM.zip › cutoff60_111taxa/cutoff60_newRIPPS-included_111taxa/html_content/networks/2023-05-02_09-52-12_hybrids_glocal_lichens_c0.60/NRPS/index.html]

BiG-SCAPE Result


Loading data...
